# A novel homozygous frameshift mutation in the *CCDC151* causing primary ciliary dyskinesia with azoospermia in a Chinese family

**DOI:** 10.3389/fmed.2026.1929775

**Published:** 2026-09-23

**Authors:** Hui Fan, Lin Wang, Xianglin Zhou, Xiangyang Lu, Binyi Yang, Lv Liu, Danhui Yang, Ting Guo, Hong Luo

**Affiliations:** 1 Department of Pulmonary and Critical Care Medicine, The Second Xiangya Hospital, Central South University, Changsha, Hunan, China; 2 Research Unit of Respiratory Disease, Central South University, Changsha, Hunan, China; 3 Clinical Medical Research Center for Pulmonary and Critical Care Medicine in Human Province, Changsha, Hunan, China; 4 Hunan Diagnosis and Treatment Center of Respiratory Disease, Changsha, Hunan, China; 5 Changsha Human Ciliary Disease and Translational Medicine Science and Technology Innovation Center, Changsha, Hunan, China

**Keywords:** azoospermia, CCDC151, frameshift variant, infertility, primary ciliary dyskinesia

## Abstract

**Background:**

CCDC151 is essential for outer dynein arms (ODA) docking and assembly, and loss of its function impairs mucociliary clearance, leading to primary ciliary dyskinesia (PCD). Pathogenic variants in *CCDC151* have been reported rarely, and azoospermia associated with *CCDC151*-related PCD remains poorly characterized**.**

**Methods:**

Whole exome sequencing (WES) and sanger sequencing were used to identify disease-related genes in a Chinese family with PCD. Domain analysis was applied to predict the potential impact of the variant on CCDC151. Respiratory multiciliated cells were examined by high speed video microscopy analysis (HSVA), transmission electron microscopy (TEM), and immunofluorescence (IF) staining to assess the structural and functional consequences of the identified variant. Functional assays in Human embryonic kidney 293 T (HEK293T) cells were performed to assess the impact of the identified variant on mRNA and protein expression.

**Results:**

We described a 40-year-old man with recurrent respiratory infections, chronic sinusitis, diffuse bilateral bronchiectasis, and azoospermia. WES identified a previously unreported homozygous frameshift variant in *CCDC151* (NM_145045.5:c.986dupT; p. Gln330ThrfsTer40), predicted to result in premature protein truncation. IF staining showed complete loss of the ODA components DNAH5 and DNAI1 from respiratory cilia, and TEM confirmed absence of ODA in the cilia axonemes. Consistently, HSVA revealed complete ciliary immotility. Functional assays revealed comparable mRNA levels between wild-type and mutant constructs, and western blotting detected a truncated protein of approximately 41 kDa in mutant-transfected cells, confirming that the c.986dupT variant produces a stable truncated CCDC151 protein. These findings support the pathogenicity of this novel *CCDC151* variant. We further reviewed published cases of *CCDC151*-related PCD, highlighting the rarity and allelic heterogeneity of this subtype.

**Conclusion:**

We identified a novel homozygous frameshift *CCDC151* variant in a patient with PCD and azoospermia, expanding the mutational and phenotypic spectra of *CCDC151*-related PCD and supporting an association between CCDC151 dysfunction and azoospermia.

## Introduction

Primary ciliary dyskinesia (PCD, OMIM: 244400) is a rare genetic motile ciliopathy characterized by structural and/or functional abnormalities of motile cilia (1). The disorder predominantly follows an autosomal recessive inheritance pattern, with rare autosomal dominant or X-linked forms. Epidemiological studies estimate a global prevalence of approximately 1/7554 (2). To date, more than 50 genes have been implicated in PCD pathogenesis (3), most encoding structural components of the ciliary axoneme, including dynein arms, radial spokes, and associated docking complexes. Defects in these genes disrupt ciliary assembly and motility, leading to impaired mucociliary clearance across multiple organ systems. The classic clinical presentation includes chronic respiratory infections, laterality defects, and subfertility (4). Kartagener syndrome, defined by the triad of bronchiectasis, chronic sinusitis, and situs invs., represents a distinct phenotypic subgroup of PCD.

The *CCDC151* gene, located at chromosome 19q13.32, encodes a 595-amino acid protein rich in coiled-coil domains. Functional impairment of CCDC151 results in ODA deficiency and consequent ciliary immotility. Studies across several model organisms have established that CCDC151 is critically required for the proper assembly and stable docking of outer dynein arms (ODAs) onto the peripheral microtubule doublets of the ciliary axoneme. In 2014, Hjeij et.al first identified biallelic mutations in *CCDC151* as a cause of PCD in humans (5), with affected individuals displaying chronic respiratory symptoms and situs abnormalities. However, despite the growing list of PCD-associated genes, cases attributable to *CCDC151* mutations remain exceptionally rare, with fewer than twenty families reported to date. Azoospermia has never been reported in patients with *CCDC151* mutations, despite the fact that male infertility is a well-known symptom of PCD that is linked to decreased sperm flagellar motility.

Here, we report a novel homozygous frameshift mutation in *CCDC151* gene identified in a patient with PCD and azoospermia. Through structural and motility analyses, we confirmed that this loss-of-function variant disrupts the “9 + 2” axonemal structure, resulting in typical ODA defects and loss of ciliary motility.

## Methods

### Patient

The patient was recruited from a cohort of individuals with suspected inherited bronchiectasis at the Second Xiangya Hospital of Central South University, Changsha, China. The patient's clinical history, laboratory tests, and radiographic data were collected. This study was approved by the Ethics Committee of the Second Xiangya Hospital and was conducted in accordance with institutional and international ethical standards. Written informed consent was obtained before participation.

### DNA extraction, whole exome sequencing (WES) and sanger sequencing

Genomic DNA was isolated from peripheral blood samples obtained from the proband using the QIAamp DNA Blood Mini Kit (QIAGEN, Valencia, CA). Exome capture was performed with the Agilent SureSelect Human All Exon V5 Kit (Agilent, California, USA) followed by sequencing on an Illumina HiSeq 4000 platform (Illumina Inc., San Diego, USA). The mean target coverage was 215×, and 99.27% of the target region was covered. Sequencing reads were aligned to the human reference genome (GRCh37/hg19) by the Burrows Wheeler Aligner software. ANNOVAR is used to do annotation for Variant Call Format file. Identified variants were classified based on the American College of Medical Genetics and Genomics (ACMG) guidelines into five categories. Variant filtering was conducted with the following steps (Figure 1): (1) Variants (minor allele frequency > 0.01) recorded in the 1000 Genomes Project, Exome Sequencing Project (ESP), Genome Aggregation Database (gnomAD), and Novo in-house Database were excluded; (2) Exonic and splice-site variants were retained; (3) The functional impact of SNVs was predicted using bioinformatics tools including MutationTaster, PolyPhen-2, SIFT, CADD, and LoFtool; (4) Homozygous variants were retained to be filtered by a PCD-related gene panel. Mutation was validated by Sanger sequencing with the following primers: forward, 5’-GCCCGGGTGCCTTTATC-3’; reverse, 5’-AGATCACCTCCATCTGGTACAT-3’.

**Figure 1:**
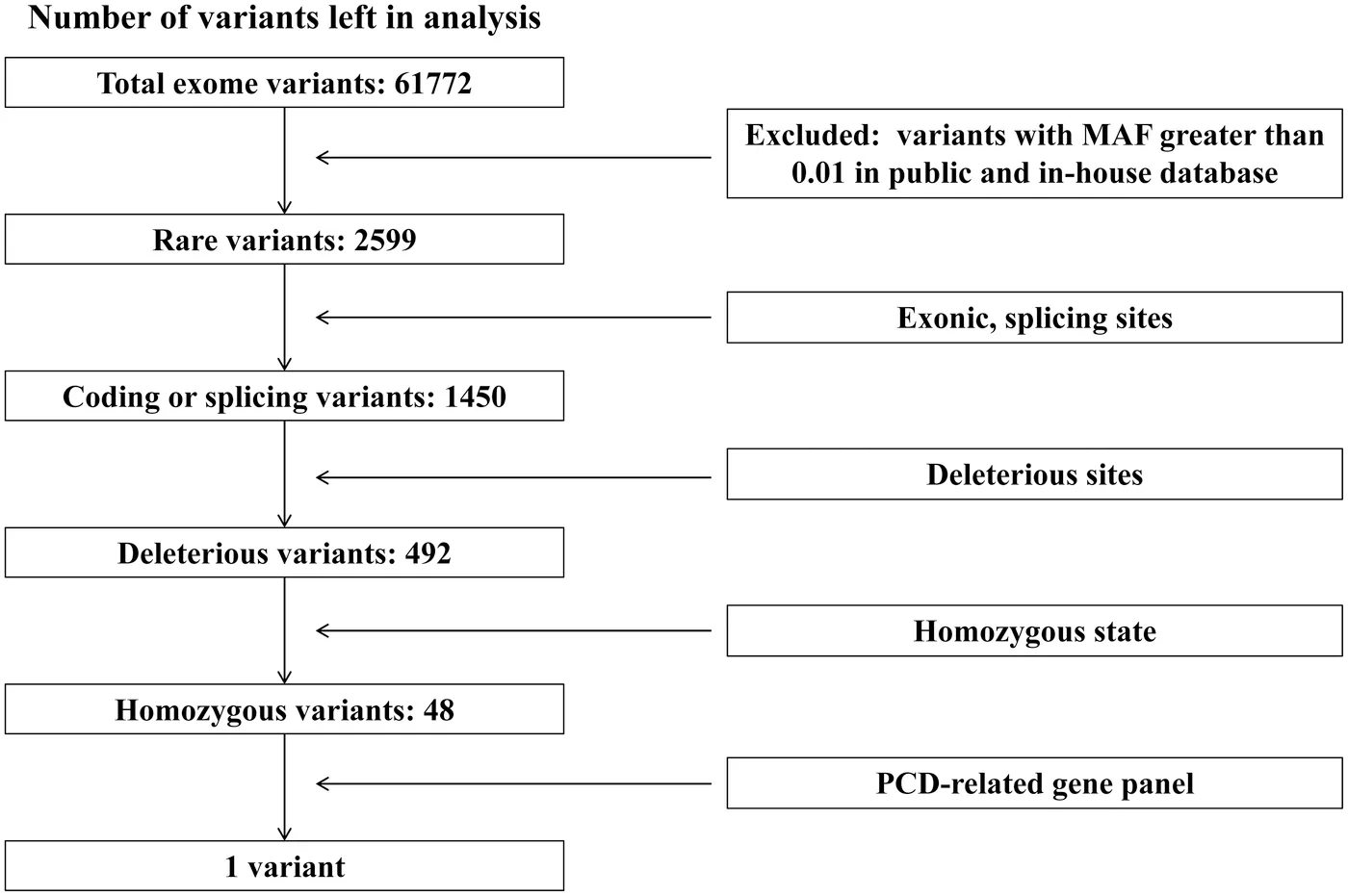
Workflow of the variant filtering strategy.

### Immunofluorescence (IF) staining

Nasal epithelial tissues were collected from the proband and healthy control and subsequently fixed in 4% paraformaldehyde at 4℃. IF staining was performed as described previously (6). In brief, tissues were incubated overnight at 4℃ with primary antibodies targeting the following proteins: DNAH5 (HPA037470, 1:100, Sigma-Aldrich, DNAI1 (12756-1-AP, 1:25, Proteintech), DNALI1 (PA5-97673, 1:25, Thermo) and anti-α-tubulin (T9026, 1:500, Sigma-Aldrich). After washing, tissues were incubated at 37℃ for 2 h with corresponding secondary antibodies: Alexa Fluor 488 anti-mouse IgG (ab150113, Abcam, 1:500) and Alexa Fluor 555 anti-rabbit IgG (A31572, Invitrogen, 1:500), and then were stained with DAPI at room temperature for 5 min. Fluorescence signals were photographed using an Olympus BX53 fluorescence microscope and analyzed using the cellSens Dimension software.

### Transmission electron microscopy (TEM)

Nasal epithelial samples were processed for ultrastructural analysis according to established protocols (7). Briefly, sample was fixed with glutaraldehyde and osmium tetroxide, post-fixed with sucrose and osmium oxide, and dehydrated with gradient ethanol. Ultrathin sections were prepared and further contrasted with uranyl acetate and lead citrate. The sections were examined under a Hitachi HT7700 transmission electron microscope equipped with a MegaView III digital camera (Olympus Optical).

### High speed video microscopy analysis (HSVA)

Nasal epithelial samples from proband were photographed using an upright Olympus BX53 microscope (BX53, Olympus) equipped with a 40× objective lens and semiconductor camera (Prime BSI^TM^, Photometrics) at a rate of 500 fps at 37℃. Ciliary beat frequency was quantified using the validated open source software (CiliarMove).

### Plasmids construction

Wild-type and mutant CCDC151 expression plasmids were custom-synthesized by a commercial biotechnology company (Bochu Biotechnology, Changsha, China). The wild-type construct contained the full-length coding sequence of human CCDC151 (NM_145045.5), while the mutant construct carried the c.986dupT frameshift variant (p. Gln330ThrfsTer40). Both constructs were cloned into the pcDNA3.1 vector with an N-terminal 3×Flag tag. An empty pcDNA3.1 vector containing the same 3×Flag tag was used as a negative control.

### Cell culture and transfection

Human embryonic kidney 293 T (HEK293T) cells (GNHu17, Shanghai, China) were maintained in Dulbecco's modified Eagle's medium (DMEM, Gibco, USA) supplemented with 10% fetal bovine serum (Gibco, USA) and 1% penicillin-streptomycin (Gibco, USA) at 37 °C in a 5% CO₂ humidified incubator. For transfection, cells were seeded into 6-well plates at a density of 4 × 10⁵ cells per well and cultured overnight to reach 70%–80% confluence. Transfection was performed using Lipofectamine 3000 reagent (L3000015, Thermo Fisher Scientific, USA) according to the manufacturer's protocol. Each well received 2.5 μg of plasmid DNA (wild-type, mutant, or empty vector) and 5 μL of P3000 reagent diluted in Opti-MEM (Gibco, USA). Total RNA was harvested at 36 h post-transfection, and protein lysates were collected at 48 h post-transfection.

### RNA extraction and reverse transcription quantitative real-time polymerase chain reaction (RT qPCR)

Total RNA was extracted using an RNA extraction kit (ER501-01-V2, Full Gold, China). The reverse transcription of mRNA to complementary DNA was performed with a a PrimeScript RT reagent kit (M1632, Thermo Fisher Scientific, USA). RT qPCR was carried out with SYBR Green Master Mix (AG11718, Accurate Biology, China) on a 7500 Fast Real-Time PCR System (Applied Biosystems, USA). The primers for CCDC151 were: forward 5′-TACCTAGAGGAGACCTTGGTTCG-3′ and reverse 5′-GATGGTGTCGTCGGACTGTA-3′. GAPDH was used as an endogenous control with primers forward 5′-GGAGCGAGATCCCTCCAAAAT-3′ and reverse 5′-GGCTGTTGTCATACTTCTCATGG-3′. Relative mRNA expression levels were calculated using the 2⁻*^ΔΔ^*^Ct^ method.

### Western blot analysis

Proteins were separated on a sodium dodecyl sulfate-polyacrylamide gel, transferred onto a polyvinylidene fluoride membrane, and blocked with 5% non-fat milk at room temperature for 1 h, followed by incubation with indicated primary antibodies overnight at 4 °C. The primary antibodies used were: anti-Flag monoclonal antibody (F1804, 1:3000, Sigma-Aldrich) and anti-GAPDH (10494-1-AP, 1:2000, Proteintech). The next day, the membrane was washed with Tris-buffered saline containing Tween-20 and then incubated with horseradish peroxidase (HRP)-conjugated secondary antibody (RGAM009, 1:10000, Proteintech) at room temperature for 1 h. Finally, the membranes were developed with enhanced chemiluminescence detection reagents.

### Statistical analysis

Statistical analyses were performed using GraphPad Prism (version 9.5.0). For RT-qPCR, six independent transfection replicates were used to assess experimental consistency, and the data are presented as individual data points with summary statistics (mean ± SD). For Western blotting, two independent experiments were performed, and representative blots are shown.

## Results

### Clinical characteristics of the patient

A 40-year-old man from a non-consanguineous family (Figure 2A), who reported no history of paternity consistent with his azoospermia diagnosis, presented with a lifelong history of recurrent productive cough, chronic nasal obstruction, persistent rhinorrhea, and bilateral hearing impairment. Nasal nitric oxide (nNO) was markedly reduced to 37 nL/min, below the diagnostic threshold for PCD. Chest high resolution computed tomography (HRCT) revealed bilateral, lower-lobe-predominant bronchiectasis, and paranasal sinus CT showed chronic sinusitis, with no evidence of laterality defects (Figure 2B). Semen analysis demonstrated azoospermia (no spermatozoa detected in the pellet after centrifugation at 3000 rpm for 15 min), semen volume and pH were within normal ranges (Table 1). The patient declined additional evaluations, including testicular ultrasonography and serum hormone profiling. HSVA of nasal epithelial samples obtained by brush biopsy revealed complete ciliary immotility (Supplementary Video).

**Figure 2:**
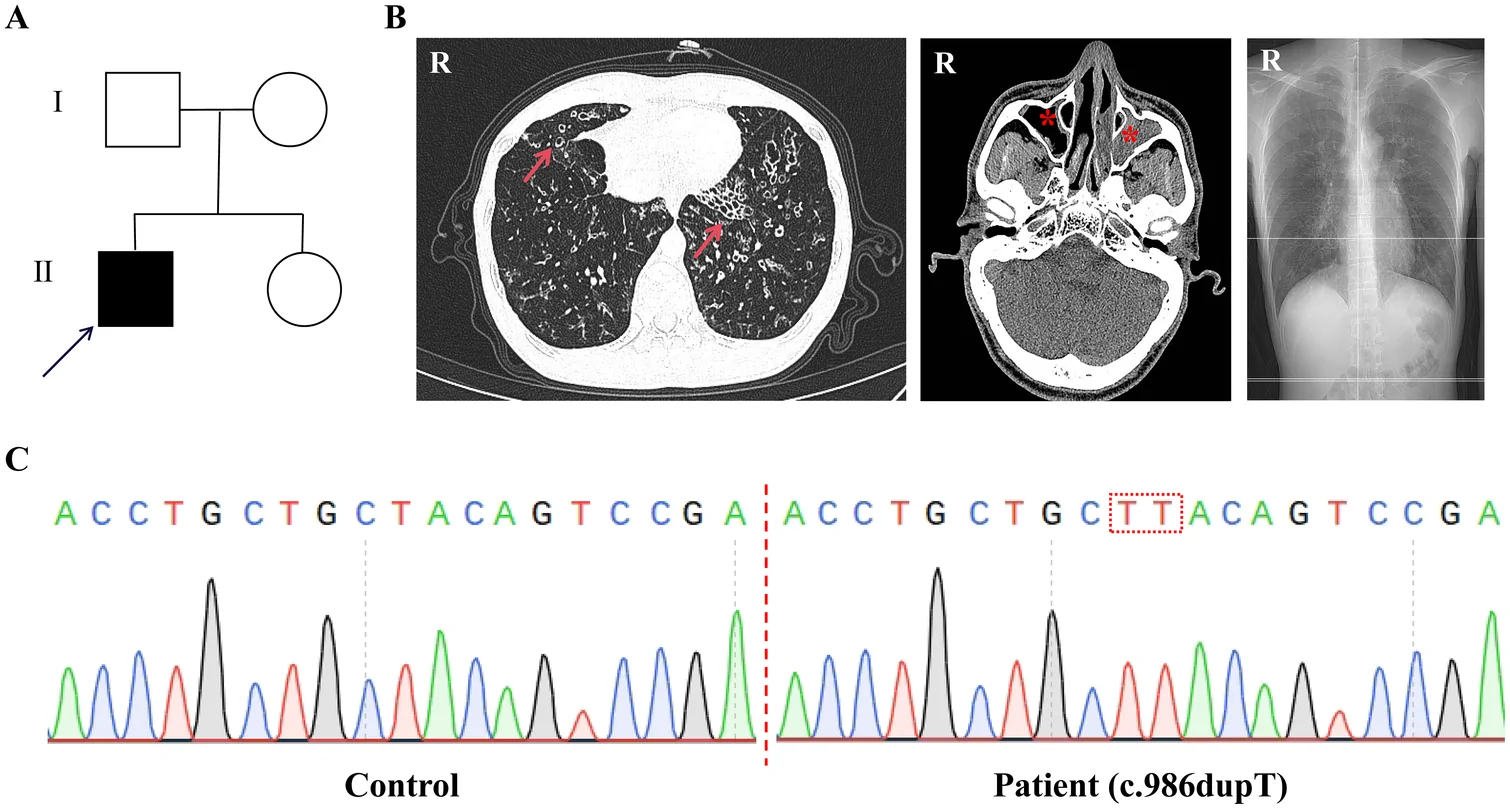
Clinical data and genetic analysis of the patient. **(A)** Pedigree of the patient. The proband (Ⅱ-1) is indicated by a black arrow. **(B)** The computed tomography scan show bilateral bronchiectasis (red arrows) and sinusitis (red asterisks), but no evidence of laterality defects was identified. **(C)** Sanger sequencing confirmed the *CCDC151* variant identified in the patient.

**Table 1:** Semen analysis results of the patient.

Parameter	Patient	Normal reference (WHO-5)
Semen volume (mL)	2.5	≥1.5
Liquefaction time (min)	30	≤60
Semen pH	7.3	≥7.2
Sperm concentration (×10⁶/mL)	No spermatozoa were observed after centrifugation	≥15
Progressive motility (%)	/	≥32
Sperm motility (%)	/	≥40

### Identification of a novel *CCDC151* variant

To elucidate the genetic etiology of the patient's condition, WES was performed on genomic DNA from the patient. A total of 61772 exonic variants were identified, 2599 rare variants were left by excluding the variants with a minor allele frequency (MAF) > 0.01 in public databases mentioned in methods. After retaining only coding or splicing variants, 1450 variants remained. These were further filtered for predicted deleteriousness using multiple in silico tools, yielding 492 variants. Subsequently, 48 variants in a homozygous state were selected, and then filtered by the PCD-related gene panel based on the clinical phenotype of the patient. A homozygous frameshift variant of *CCDC151* (NM_145045.5:c.986dupT; p. Gln330ThrfsTer40) was identified in the sample of patient finally. The variant was classified as pathogenic according to the American College of Medical Genetics and Genomics (ACMG) criteria (PVS1, PM2, PP3, and PP4) (8). Sanger sequencing confirmed the homozygous status of this variant (Figure 2C).

### Structural prediction and functional validation of CCDC151 mutation-induced defects

Homology modeling using the AlphaFold Server predicted significant structural consequences of this mutation (Figure 3A). Compared to the wild-type protein, the mutant protein lacks the entire third coiled-coil domain due to premature truncation, including critical C-terminal functional regions essential for proper protein function (Figure 3B).

**Figure 3:**
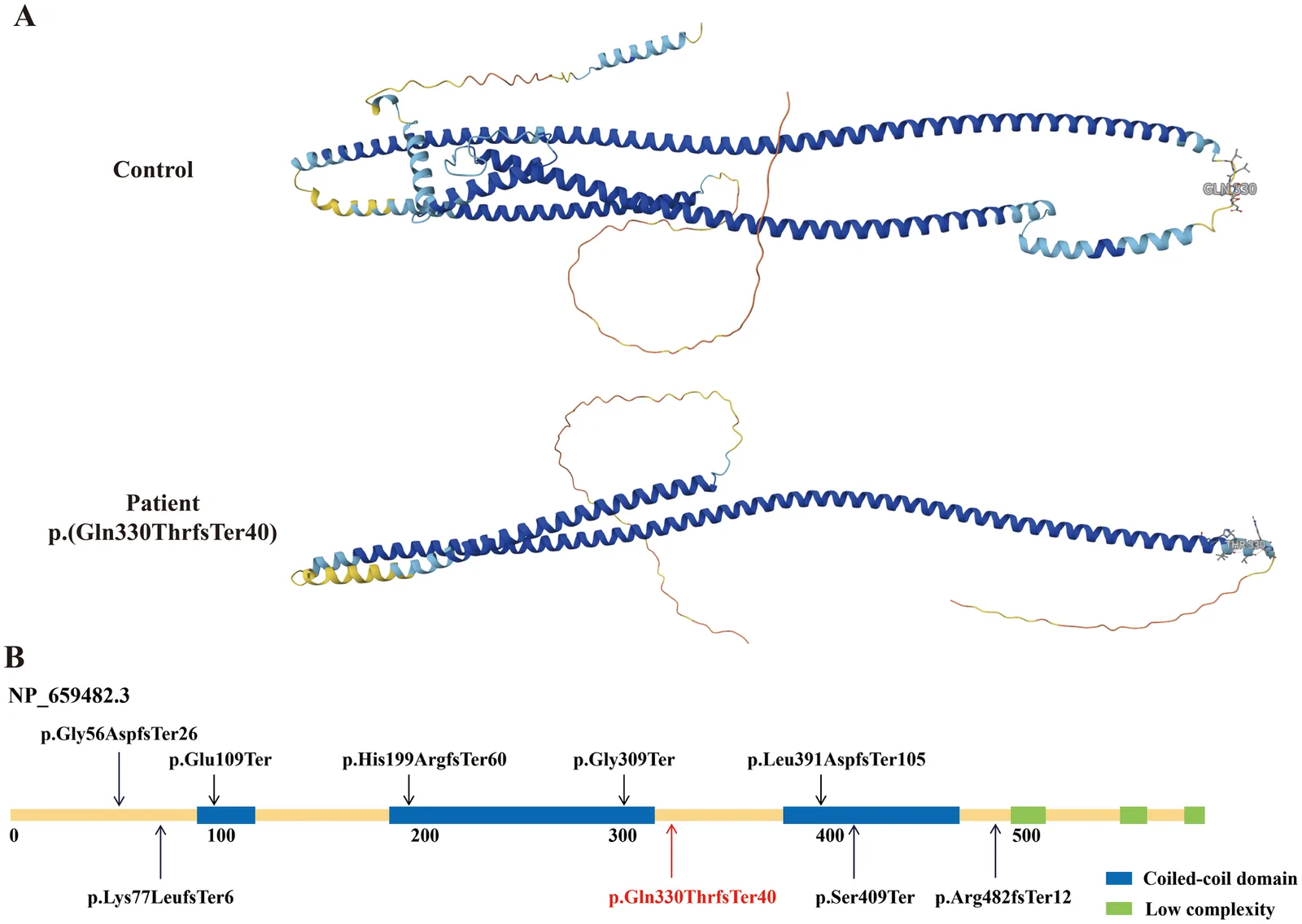
Predicted effects of the *CCDC151* variant and distribution of reported CCDC151 protein alterations. **(A)** AlphaFold predicted structures of normal CCDC151 (NP_659482.3) and the p.(Gln330ThrfsTer40) variant identified in this study. **(B)** Domain organization of CCDC151 showing previously reported protein-altering variants and the variant from this study.

To validate these bioinformatic predictions, TEM was employed to examine the ciliary ultrastructure in the patient's nasal epithelial mucosal tissue. TEM analysis (Figure 4A) revealed extensive absence of ODA in the ciliary axonemes, while inner dynein arms (IDA) and the “9 + 2” microtubule arrangement remained largely intact. IF staining was performed, showing complete absence of DNAH5 and DNAI1 (Figure 4B), representing the heavy and intermediate chain components of the ODA, respectively. In contrast, DNALI1, a light intermediate chain of IDA, demonstrated normal localization patterns (Figure 4B). These results provide functional evidence that the mutation disrupts the biological role of CCDC151 as a crucial component of the ODA-docking complex, specifically affecting ODA assembly while preserving IDA integrity.

**Figure 4:**
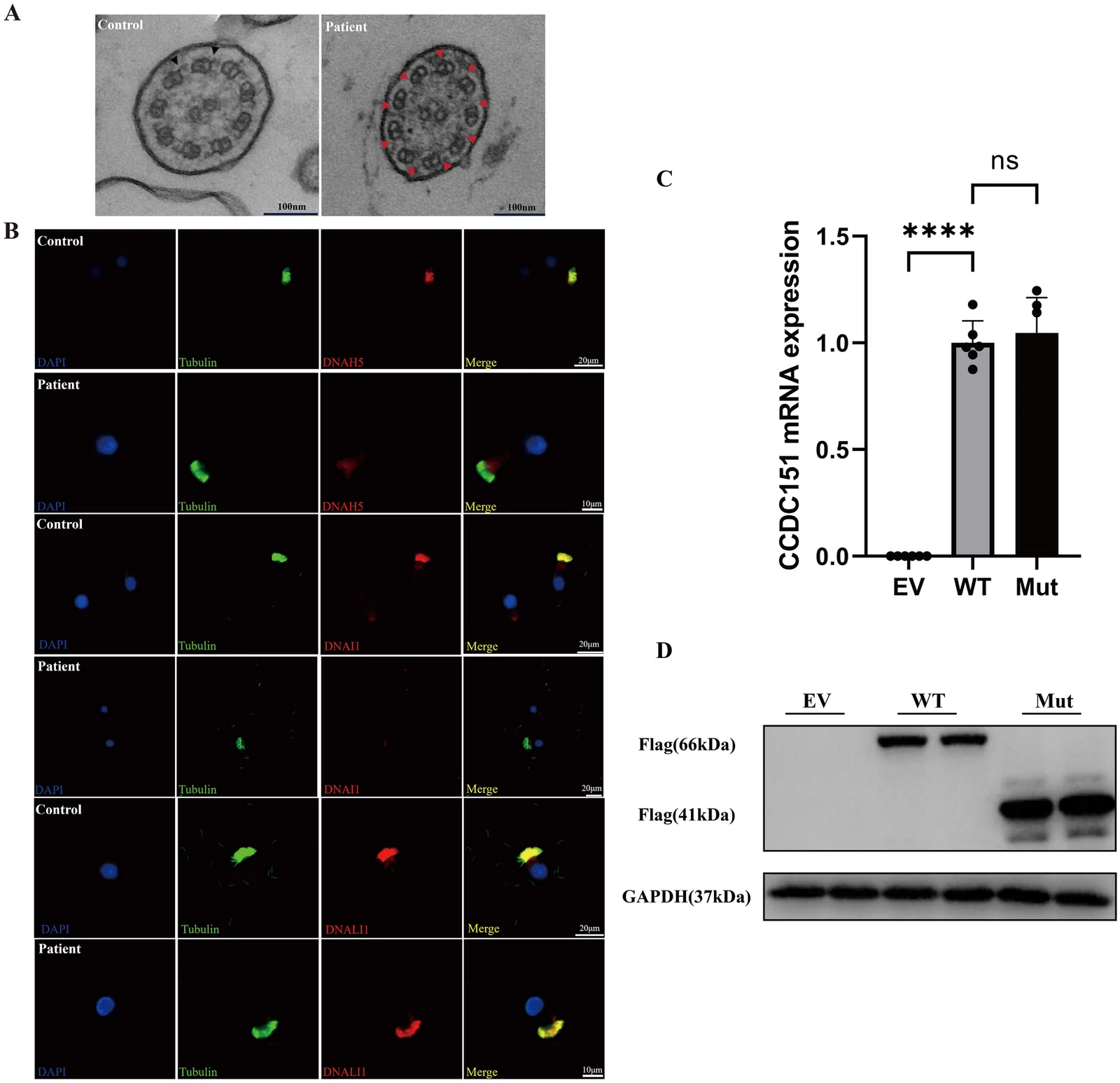
*CCDC151* mutation result in outer dynein arms defects and produces a truncated protein. **(A)** Transmission electron microscopy shows loss of outer dynein arms (ODAs) in patient respiratory cilia. Black arrows indicate ODAs; red arrows indicate absent ODAs. **(B)** Immunofluorescence shows loss of ODA markers DNAH5 and DNAI1, with preserved DNALI1 staining in patient cilia. **(C)** RT-qPCR analysis of CCDC151 mRNA expression in HEK293T cells. Cells were transiently transfected with empty vector (EV), wild-type (WT), or mutant (Mut) CCDC151 expression plasmids. Both WT and Mut constructs resulted in robust CCDC151 mRNA levels compared with the EV control (****p < 0.0001). No significant difference in mRNA expression was observed between the WT and Mut groups (ns). Each dot represents one independent culture replicate derived from donor-specific primary cells. Data are shown as mean ± SD. **(D)** Western blot analysis of Flag-tagged CCDC151 protein expression in transfected HEK293T cells. Full-length CCDC151 (66 kDa) is detected in WT-transfected cells, while a smaller truncated protein (41 kDa) is observed in Mut-transfected cells. No signal is detected in EV control. GAPDH serves as a loading control.

To elucidate the molecular consequence of the c.986dupT variant, we performed RT-qPCR and Western blotting in HEK293T cells transfected with wild-type, mutant, or empty vector plasmids. RT-qPCR revealed comparable mRNA levels between wild-type and mutant constructs, excluding nonsense-mediated mRNA decay as the underlying mechanism (Figure 4C). Western blotting with anti-Flag antibody detected a band corresponding to full-length CCDC151 in wild-type cells, whereas a smaller band matching the predicted size of the truncated protein was observed in mutant-transfected cells. No signal was detected in the empty vector control (Figure 4D). These findings confirm that the c.986dupT variant produces a stable truncated protein lacking the third coiled-coil domain and critical C-terminal regions required for ODA docking.

## Discussion and conclusion

In this study, we report a case of PCD attributable to a novel homozygous mutation in the *CCDC151* gene. The patient exhibited the classical respiratory phenotype of PCD, including chronic pansinusitis and lower-lobe–predominant bronchiectasis, and was accompanied by azoospermia.

PCD is a genetically heterogeneous ciliopathy caused by structural and/or functional defects of motile cilia, ultimately impairing mucociliary clearance and leading to chronic sino-pulmonary disease, conductive hearing impairment, and variable reproductive dysfunction. Motile cilia possess a highly conserved “9 + 2” axonemal architecture, and their coordinated oscillatory motion relies primarily on the activity of the IDA and ODA. As described above, ODA assembly and docking are critical for ciliary motility, and disruption of these processes is a well-established cause of PCD. Indeed, the majority of genetically defined PCD cases to date arise from defects in ODA assembly or stability. CCDC151 is a critical component of the ODA docking complex, and its dysfunction leads to ODA deficiency. Beyond this well-characterized role, a functional link between CCDC151 and the intraflagellar transport (IFT) system has been suggested: CCDC151 is specifically expressed in IFT-dependent motile cilia, and its depletion impairs dynein arm assembly and ciliary localization of docking components (9). Thus, while ODA docking remains the primary defect in CCDC151 deficiency, a concomitant disturbance in IFT-mediated trafficking of dynein components cannot be excluded.

To date, nine pathogenic CCDC151 mutations have been reported (Figure 3B): three nonsense mutations (p. Gly309Ter, p. Ser409Ter, p. Glu109Ter), five frameshift mutations (p. Gly56AspfsTer26, p. Lys77LeufsTer6, p. His199ArgfsTer60, p. Leu391AspfsTer105, p. Arg482fsTer12), and one splicing mutation (c.244 + 1G > A) (5, 10–18). Consistent with these previously reported mutations, the c.986dupT variant identified in our patient is also predicted to disrupt evolutionarily conserved coiled-coil domains essential for ODA-docking complex assembly, thereby impairing ODA docking and causing ciliary immotility. Beyond this shared mechanism, our functional assays revealed that this frameshift variant produces a stable truncated protein lacking the third coiled-coil domain and critical C-terminal regions, as demonstrated by comparable mRNA levels and detection of a truncated protein by Western blotting. Thus, this variant results in a structurally compromised CCDC151 protein rather than complete loss of expression.

Sperm flagella share similar axonemal organization and dynein arm dependency as airway cilia, pathogenic *CCDC151* variants would be anticipated to affect sperm motility. Indeed, male infertility is a well-recognized but often undercharacterized component of PCD. Defects in genes encoding axonemal, dynein arm, and other ciliary components can lead to a broad spectrum of sperm abnormalities, ranging from severe asthenozoospermia and multiple morphological abnormalities of the flagella (MMAF) to complete sperm immotility, oligozoospermia, and even azoospermia (19). *Ccdc151*-deficient mice exhibit severe asthenoteratozoospermia with multiple morphological abnormalities of the sperm flagella and can also present as azoospermia. Analysis of seminiferous tubules in *Ccdc151*-deficient mice revealed impaired spermatogenesis, with an accumulation of tubules at the spermiogenesis and spermiation stages (20), which suggests that disruption of ciliary proteins can compromise sperm transport. Despite these findings in animal models, infertility in human males with CCDC151 mutations has not yet been documented (Table 2). This may be attributable to the limited number of reported cases, the generally young age of diagnosed individuals, or incomplete reproductive evaluation. Moreover, emerging evidence of reduced female fertility in CCDC151-related PCD further underscores the need for comprehensive reproductive assessment in affected individuals (16). Although spermatogenic defects are clearly observed in murine models, caution is warranted when translating these findings to human azoospermia, given well-known species differences in reproductive tract anatomy and ciliary function. Unfortunately, our patient declined further diagnostic procedures; therefore, the exact mechanism underlying his azoospermia remains to be elucidated. One possibility is obstructive azoospermia resulting from impaired ciliary beating in the efferent ductules, where motile cilia are essential for luminal fluid mixing and sperm transport. Alternatively, primary spermatogenic defects may also contribute, as supported by findings in *Ccdc151*-knockout mice, which showed accumulation of seminiferous tubules at the spermiogenesis/spermiation stages, with round spermatids failing to elongate and being subsequently unable to release into the tubular lumen.

**Table 2:** Clinical findings of individuals of previous reported *CCDC151*-associated PCD.

ID	Variants	Sex	Age at diagnosis	nNO	NRD	Bronchiectasis	Sinusitis	Situs Inversus	Subfertility	Ref
1	c.925G > T, p. Glu309Ter	M	16y	NR	Yes	Yes	Yes	Yes	NR	(5)
2	c.925G > T, p. Glu309Ter	F	10w	9–11ppb	Yes	Yes	Yes	Yes	NR	
3	c.925G > T, p. Glu309Ter	F	1y	NR	Yes	NR	Yes	Yes	NR	
4	c.925G > T, p. Glu309Ter	M	6y	NR	Yes	NR	NR	Yes	NR	
5	c.1256C > A, p. Ser419Ter	M	12y	NR	No	NR	Yes	Yes	NR	
6	c.925G > T, p. Glu309Ter	M	NR	5^a^	NR	Yes	Rhinitis	NR	NR	(10)
7	c.325G > T, p. Glu109Ter	M	16y	1.2nL/min	No	Yes	Yes	Yes	NR	(11)
8	c.583_595dupGCGCAAAACAGAC, p. His199ArgfsTer60	NR	NR	18nL/min	NR	NR	NR	Yes	NR	(12)
9	c.925G > T, p. Glu309Ter	F	6y	NR	No	NR	Rhinitis	Yes	NR	(13)
10	c.925G > T, p. Glu309Ter	F	9m	NR	Yes	NR	Rhinorrhea	Yes	NR	
11	c.167delG, p. G56DfsTer26	M	34y	NR	No	Yes	Yes	Yes	NR	(14)
12	c.229_233delinsCTCAGGTATATAACATAAACTCAGTTACATAA, p. Lys77LeufsTer6	M	7y	Low	Yes	NR	NR	No	NR	(15)
13	c.1166_1169dupAGAC, p. Leu391AspfsTer105	F	35y	6nL/min	NR	Yes	Yes	Yes	Yes	(16)
14	c.1445_1446delGC, p. Arg482fsTer12	F	NR	NR	Yes	Yes	Yes	Yes	NR	(17)
15	c.1445_1446delGC, p. Arg482fsTer12	F	NR	NR	Yes	Yes	Yes	Yes	NR	
16	c.244 + 1G > A	M	30y	NR	NR	Yes	NR	Yes	NR	(18)

M, male; F, female; nNO, nasal nitric oxide; NR, not report; NRD, neonatal respiratory distress.

a The data is presented as unitless quantity.

Several limitations of this study should be acknowledged. First, parental DNA samples were unavailable, preventing formal segregation analysis to confirm the variant's inheritance pattern. Second, comprehensive reproductive investigations, including testicular ultrasonography and hormonal profiling, could not be performed as the patient declined further testing, limiting our ability to determine the exact mechanism underlying his azoospermia. Despite these limitations, the combined genetic, ultrastructural, and functional evidence strongly supports the pathogenicity of the identified variant.

Collectively, this finding represents the first case of azoospermia associated with a *CCDC151* mutation, enriching current understanding of the phenotypic and genotypic spectrum of *CCDC151*-associated PCD. The identification of a novel homozygous *CCDC151* mutation further highlights the importance of integrating genetic testing with ciliary functional assessment in patients with suspected PCD, particularly in the absence of laterality defects. Continued investigation into the mechanistic roles of CCDC151 in ODA assembly, as well as systematic evaluation of reproductive function in affected patients, will be crucial for refining genotype–phenotype correlations and improving clinical management strategies.

## Data Availability

The datasets presented in this article are not readily available due to privacy concerns. Requests to access the datasets should be directed to the corresponding authors.
